# Strong effects of coral species on the diversity and structure of reef fish communities: A multi-scale analysis

**DOI:** 10.1371/journal.pone.0202206

**Published:** 2018-08-13

**Authors:** Valeriya Komyakova, Geoffrey P. Jones, Philip L. Munday

**Affiliations:** 1 ARC Centre of Excellence for Coral Reef Studies, and College of Marine and Environmental Sciences, James Cook University, Townsville, QLD, Australia; 2 Department of Zoology, University of Melbourne, Melbourne, VIC, Australia; Department of Agriculture and Water Resources, AUSTRALIA

## Abstract

While there is increasing evidence for habitat specialization in coral reef fishes, the extent to which different corals support different fish communities is not well understood. Here we quantitatively assess the relative importance of different coral species in structuring fish communities and evaluate whether sampling scale and coral colony size affect the perceived strength of fish-habitat relationships. Fish communities present on colonies of eight coral species (*Porites cylindrica*, *Echinopora horrida*, *Hydnophora rigida*, *Stylophora pistillata*, *Seriatopora hystrix*, *Acropora formosa*, *A*. *tenuis* and *A*. *millepora*) were examined in the Lizard Island lagoon, Great Barrier Reef, Australia. Additionally, the differences in fish communities supported by three coral species (*P*. *cylindrica*, *E*. *horrida*, *H*. *rigida*) were investigated at three spatial scales of sampling (2x2 m, 1x1 m, 0.5x0.5 m). Substantial differences in fish communities were observed across the different coral species, with *E*. *horrida* and *H*. *rigida* supporting the most fish species and individuals. Coral species explained more of the variability in fish species richness (20.9–53.6%), than in fish abundance (0–15%). Most coral species supported distinctive fish communities, with dissimilarities ranging from 50 to 90%. For three focal coral species, a greater amount of total variation in fish species richness and fish abundance was evident at a larger scale of sampling. Together, these results indicate that the structure of reef fish communities is finely tuned to coral species. Loss of preferred coral species could have profound effects on reef fish biodiversity, potentially more so than would be predicted on the basis of declining coral cover alone.

## Introduction

Habitat characteristics are known to play a key role in structuring natural communities [1–2]. In many ecosystems, living organisms create biogenic habitat that provides critical resources for a wide variety of mobile species. For example, terrestrial forest biomes form the habitat structure essential for the survival of many insects, birds and mammals [3–5]. Likewise, macro-algae and seagrasses have a similar habitat-forming role in freshwater and temperate marine ecosystems [6–7]. Numerous studies have examined relationships between the abundance of focal species and the areal cover of biogenic habitat [8–10], however, the strength of these relationships may depend on the level at which organisms discriminate among habitat types [11–12]. If focal organisms are highly specialised and discriminate among habitat-forming species, then habitat availability should be measured at the species level. In addition, the strength of the relationships between organisms and their habitat may depend on the scale of sampling [13]. Organism-habitat relationships may be obscured if inappropriate taxonomic resolution or spatial scale of sampling are applied [14–16]. Therefore, to understand population and community responses to degrading habitats, it is necessary to understand these species and scale-specific phenomena.

On coral reefs, the complex structure of scleractinian corals produces a diversity of habitat types that provide shelter, food and sites for reproduction for other reef organisms [17–19]. Coral cover is often considered the key variable that influences the abundance of coral-reliant organisms [8, 20]. However, there is increasing evidence that many organisms preferentially associate with particular coral species or coral morphologies at critical stages of their development [12, 19, 21–24]. For example, Bonin [23] demonstrated that new recruits of at least four damselfish species (*Chrysiptera parasema*, *Pomacentrus moluccensis*, *Dascylus melanurus* and *Chrosmis retrofasciatus*) had a strong preference for a limited number of *Acropora* species. Hence, measures of overall coral cover may fail to detect species-specific habitat associations that are important in explaining the diversity of reef communities.

Some studies have shown that the presence of different coral species influences the structure of fish communities and identified the characteristics of the corals likely to be responsible for these differences [21–23, 25–26]. For example, Messmer et al. [26] identified several coral species that tend to support more diverse fish communities (e.g. *Acropora nasuta* and *Seriatopora hystrix*). The physical characteristics of coral species that attract and support a high diversity and abundance of fishes may relate to the branching structure of the coral colonies [21–22, 25]. In general, structurally more complex habitats tend to support more diverse and abundant animal communities by providing a greater variety and number of refuge sites, which in turn can decrease encounter rates between competitors as well as between predators and their prey [27–29]. Consequently, structurally complex coral species are predicted to support richer and more abundant fish communities.

The observed relationships between fish diversity or abundance and the structure of the coral community may also be dependent on the spatial scale of sampling. At very small spatial scales of sampling, fish-habitat associations may appear to break down due to patchy distribution of individuals [15, 30]. In contrast, at a large spatial scale, habitat patchiness can become homogenised and other environmental characteristics, such as depth and currents, become more important in structuring fish communities [14–16, 21]. Therefore, the spatial scale of sampling of different corals could have a significant effect on the perceived relationships between the coral community structure and the structure of fish communities [31].

Understanding habitat associations of coral reef fishes is critical given the differential susceptibility of coral species to increasing temperatures and other stressors associated with climate change [23, 32–34]. A decline in the abundance of particular coral species could have significant effects on fish communities if those coral species support diverse and abundant fish assemblages. At the same time, natural and anthropogenic disturbances will tend to reduce the average size of coral colonies, which could affect the structure of local fish communities if smaller corals support fewer individuals and/or species [21, 35]. Understanding the influence of coral species, coral structural complexity and coral colony size on fish communities will assist efforts to predict the likely consequences of coral loss to reef fish communities [18, 36].

The overall aim of this study was to assess the influence of coral species, sampling scales and coral colony size in structuring reef fish communities. We compared the abundance and richness of fish assemblages at Lizard Island on the Great Barrier Reef across a range of common coral species to determine if:

1. Some coral species support more diverse and abundant fish communities than others. We hypothesised that fish diversity and abundance would be associated with coral structural complexity, with coral species that have a complex branching structure supporting more diverse and abundant fish communities than those with a less complex branching structure.
2. The spatial scale of sampling affects the observed relationships between coral species and fish species richness and abundance. We predicted that fish-coral species associations would be less evident at smaller scales, however the performance of specific coral species would remain consistent irrespective of the scale
3. Different coral species tend to accumulate fish species richness and abundance at different rates as colony size increases. We hypothesized that more structurally complex corals would accumulate fish species richness and abundance at faster rates than less structurally complex corals.

## Materials and methods

### Ethics statement

This study was conducted in accordance with Great Barrier Reef Marine Park Authority requirements for non-extractive research and was compliant with the James Cook University Code of Conduct for Research in the Great Barrier Reef. An authorisation for this limited impact, non-extractive research in the Great Barrier Reef Marine Park was obtained from James Cook University (Authorisation letter number: MBA5). This research did not involve any endangered or protected species and no animals were sampled. This study was conducted in compliance with the James Cook University Ethics Review Committee regulations (Ethics approval project number: A1124).

### Study location

The study was conducted within the lagoon of Lizard Island, northern Great Barrier Reef (14°40’S, 145°28’E), QLD, Australia between November 2006 and January 2007. The Lizard Island lagoon is relatively shallow with a maximum depth of approximately 15 meters and with the majority of reefs situated in three to six meters depth. The lagoon is sheltered from the prevailing southeast swell and has well developed reefs around its margins.

### Sampling design

#### Coral structural characteristics

To determine if some species of coral support more diverse and abundant fish communities than others, we compared fish community structure among eight of the most commonly occurring coral species in the Lizard Island lagoon: *Porites cylindrica*, *Echinopora horrida*, *Hydnophora rigida*, *Stylophora pistillata*, *Seriatopora hystrix*, *Acropora formosa*, *A*. *tenuis* and *A*. *millepora*. These species have a complex branching structure, but differ in characteristics such as average branch length, branch density, overall colony morphology, and maximum colony size. In order to provide quantitative physical and structural descriptions of the eight coral species, we conducted different physical measures on multiple colonies of each study coral species. These measurements were used to classify corals as species with high and low structural complexity. Inter-branch space of 8–16 colonies of each of the eight coral species were measured to determine if there were significant differences in physical characteristics. Additionally, the branch length was measured for the six coral species with a branching morphology: *A*. *formosa*, *E*. *horrida*, *H*. *rigida*, *P*. *cylindrica*, *S*. *hystrix* and *S*. *pistillata*. Ten random distances between branch tips and the length of ten randomly selected branches were measured to the nearest millimetre using callipers or a ruler for longer branches. Corals were randomly sampled from around the lagoon of Lizard Island.

ANOVA and Kruskal-Wallis tests were used to examine differences in branch length and inter-branch space, respectively, among the coral species. The mean of ten branch lengths (six coral species) was calculated for each coral colony before performing ANOVA. Branch length was log10 transformed to meet the assumptions of normality and homoscedasticity (Section A in S1 Supporting Information). The mean of ten inter-branch spaces (eight coral species) was also calculated for each coral colony before performing nonparametric independent samples Kruskal-Wallis test, as the data did not meet the assumptions for ANOVA (Section A in S1 Supporting Information). The analysis was performed using SPSS.

#### Fish community structure and coral species

To determine if some species of coral support more diverse and abundant fish communities than others, we compared fish community structure among the eight coral species selected for the study (listed above). A minimum of five haphazardly selected colonies of each coral species were sampled at 0.5 x 0.5 m spatial scale (Table 1). Only colonies that showed no obvious signs of disease, bleaching or partial mortality were used. The fish assemblage occupying each coral colony (up to 0.5 m above the colony) was surveyed visually for a maximum of six minutes. During the first three minutes all the larger and more obvious fishes were counted from a distance of approximately one meter. For the following three minutes the spaces between branches were carefully and systematically searched for cryptic fish species. Only individuals that appeared to use the coral head or hovered above the coral head for the entire time of the observation were recorded. Fish that swam past the coral head during the observation period were not counted. Individuals were identified to species level and a life stage for each individual was recorded (adult, juvenile, new settler).

**Table 1:** Number of coral colonies sampled at three different scales.

Coral species	Sampling scale (m) & number of colonies sampled		
	0.5 x 0.5	1 x 1	2 x 2
*Hydnophora rigida*	13	12	6
*Echinopora horrida*	12	12	9
*Porites cylindrica*	12	11	10
*Acropora formosa*	5
*A*. *tenuis*	6
*A*. *millepora*	9
*Stylophora pistillata*	11
*Seriatopora hystrix*	10

Fish species richness, total fish abundance, and fish community structure were compared among the eight coral species that were sampled at 0.5 x 0.5 m. ANOVA was used to test for significant differences in fish species richness and total fish abundance among the eight coral species. Fish species richness and fish abundance were log10 transformed to meet assumptions of normality and homoscedasticity (Section B in S1 Supporting Information). The analysis was performed using SPSS.

A similarity percentage analysis (SIMPER) and a distance-based permutational multivariate analysis of variance (PERMANOVA) on a Bray-Curtis similarity matrix were used to compare fish assemblage structure among coral species. The 16 most abundant and frequently occurring fish taxa (minimum of 4.5% contribution to the abundance and frequency of occurrence of individuals for at least one coral species) were used in the multivariate analysis. Life stages of each fish taxa (new settlers, juveniles and adults) were considered separately in the analysis. Some fish species/age groups could not be identified to species level or occurred with low frequencies or abundances, but belong to a common genus or family. The data for these fish species were pooled together to form higher classification groups: *Gobiodon*, Pomacentrids, Pomacentrid juveniles, Labrids, Other Juveniles and Other New Settlers. Further pair-wise PERMANOVA tests were conducted as a post hoc test to identify which coral species were significantly different from each other in fish community structure. The data for this analysis were fourth root transformed prior to analysis to reduce the influence of extreme values of highly abundant fish species. Unrestricted permutations of raw data (9999) and Type III sums of squares were used to generate *P*-values due to the unbalanced design.

Bootstrapped values were calculated over 100 replications per coral species. The relationship between fish assemblage and individual coral species was visually explored using a non-metric multidimensional scaling (nMDS) plot where bootstrapped values and 95% confidence intervals were calculated over 100 replications per coral species. All multivariate analyses were performed using PRIMER v.6 and PERMANOVA+ [37–38].

#### Fish community structure and spatial scales of sampling

To determine if the spatial scale of sampling influenced the relationship between fish community structure and coral species identity, single-species coral stands of *P*. *cylindrica*, *E*. *horrida* and *H*. *rigida* were surveyed using three different sized quadrats (i.e., 2x2 m, 1x1 m and 0.5x0.5m). The spatial scales were selected based on the site attached behaviour and relatively small home ranges of the majority of the encountered fish species and on the availability of the study coral stands. Due to differences in growth forms, these were the only three of the eight coral species that could be sampled at all three spatial scales. A minimum of six haphazardly selected healthy colonies of each coral species were sampled at each spatial scale (Table 1). In most instances, individual coral colonies that closely matched one of the sampling scales were chosen. The size of each coral colony was estimated with a measuring tape. In a few instances (2x2 m scale only), the area surveyed was a portion of a larger coral colony. In these instances, a 2x2 m quadrat was haphazardly placed over the coral to delineate the sample area. Each coral colony was sampled at only one spatial scale. Fish assemblages were quantified as described above. Additionally, water depth and reef zone within the lagoon were recorded for each sampled coral.

A regression tree approach [39] was used to explore and describe the relationships between coral species, fish species richness and fish abundance at the three different scales. Depth and reef zone were also included in the analysis to account for their potential effects on the fish variables. Fish abundance was log10 transformed to reduce the influence of extreme values (Section C in S1 Supporting Information). Absolute deviations were used to estimate tree branching and the size of trees was selected by cross-validation, choosing the tree with the smallest estimated predictive error. Regression tree analysis was used because it is suited to the exploration of relationships between ecological communities and multiple environmental variables and where the sampling of variables may be unbalanced, where missing values occur, or where there are non-linear relationships between the ecological community and the environmental variables [39]. The analysis was performed using the TreesPlus (S-Plus) statistical computer package [39].

#### Fish community structure and coral colony size

To determine if different coral species accumulate fish species richness and total abundance at different rates with increasing colony size we compared the relationship of these traits to colony size for the three coral species for which a range of colony sizes was available: *P*. *cylindrica*, *E*. *horrida* and *H*. *rigida*. Colony maximum height, width and length were measured to the nearest centimetre. All fish present within the colony and up to 0.5 m above it were counted. The branches of each colony were carefully searched for cryptic species.

ANCOVAs were used to test how fish species richness and fish abundance scaled with coral colony size among the three coral species. Each fish variable was considered as a dependant variable while coral species and colony size were categorical and continuous predictors, respectively. Only *P*. *cylindrica*, *E*. *horrida* and *H*. *rigida* exhibited sufficient variation in coral colony size to be included in this analysis. Fish abundance and colony size (expressed as colony average diameter) were log10 transformed to meet the assumptions of normality, homoscedasticity and linearity (Section D in S1 Supporting Information). The analysis was performed using Statistica v8.

## Results

### Coral structural characteristics

**Fig 1:**
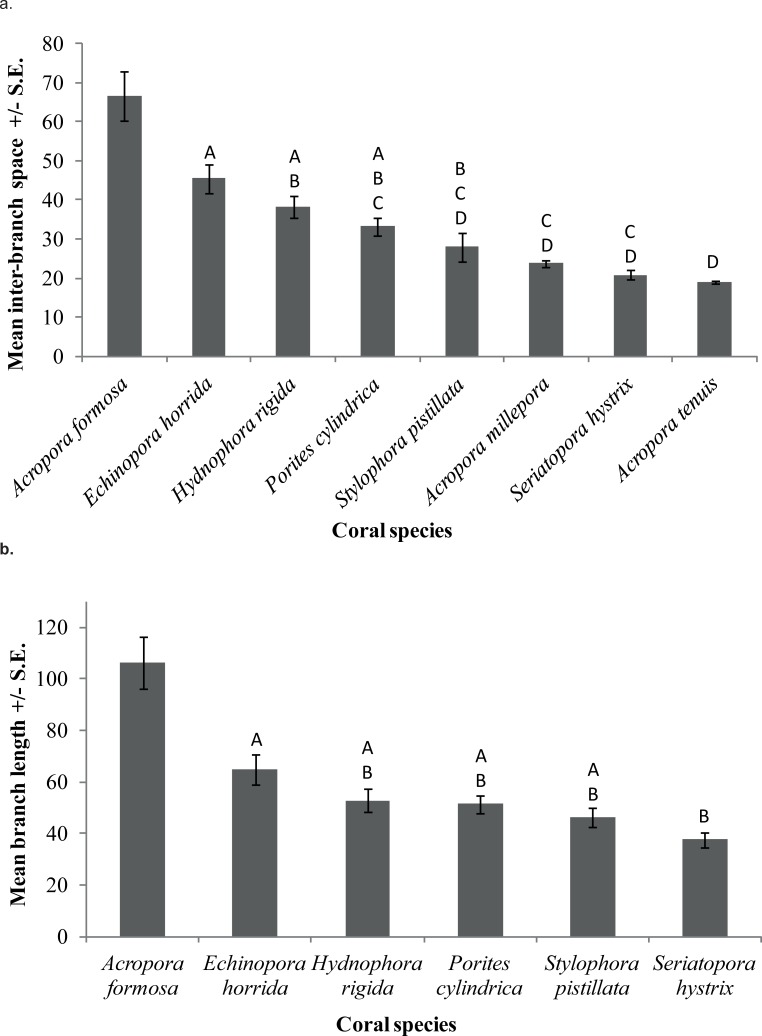
**(a)** The differences in the inter-branch space of the eight study coral species. Shared letters indicate no significant difference. **(b)** The differences in branch length of the six branching coral species. Shared letters indicate no significant difference.

The eight coral species examined differed in their average inter-branch space (Kruskal-Wallis, Chi-Square = 374.44, p < 0.001). A post hoc Tukey HSD test showed that *A*. *formosa* had significantly larger inter-branch distance than the other seven coral species ($\bar{x}$ = 66.5 mm, p < 0.05) and *A*. *tenuis* had the smallest interbranch distance among the eight studied coral species ($\bar{x}$ = 18.8 mm, p > 0.05). There was no significant difference in branch length between *A*. *tenuis*, *A*. *millepora*, *S*. *hystrix* and *S*. *pistillata* (p > 0.05). *P*. *cylindrica*, *H*. *rigida* and *E*. *horrida* had an intermediate branch length. The branch length of *P*. *cylindrica* and *H*. *rigida* also did not differ significantly from *S*. *pistillata* ($\bar{x}$ = 27.9 mm, p > 0.05) (Fig 1A).

The six branching corals differed significantly in their branch length (ANOVA, F_5,87_ = 13.815, p < 0.001). The Tukey HSD test showed that *A*. *formosa* had significantly longer branches than the other five coral species ($\bar{x}$ = 106.2 mm, p < 0.05). *S*. *hystrix* had the shortest branch length ($\bar{x}$ = 37.5 mm). The branch length of *S*. *hystrix* was significantly shorter than *E*. *horrida* ($\bar{x}$ = 64.9 mm) and *A*. *formosa*, but not the other four coral species (Fig 1B).

For the purposes of this study, corals with intermediate branch length and inter-branch distance (*E*. *horrida*, *H*. *rigida*, *P*. *cylindrica*) were considered more structurally complex than corals with only small inter-branch distance and branch length or corals with only large inter-branch distance and branch length. This definition was adopted in accordance with the assumption that intermediate inter-branch distance and branch length should provide refuge for small and medium size fishes, while excluding larger predators, similarly to the idea that lays behind the “*intermediate disturbance hypothesis”* [40].

### Fish community structure and coral species

#### Fish species richness & fish abundance

**Fig 2:**
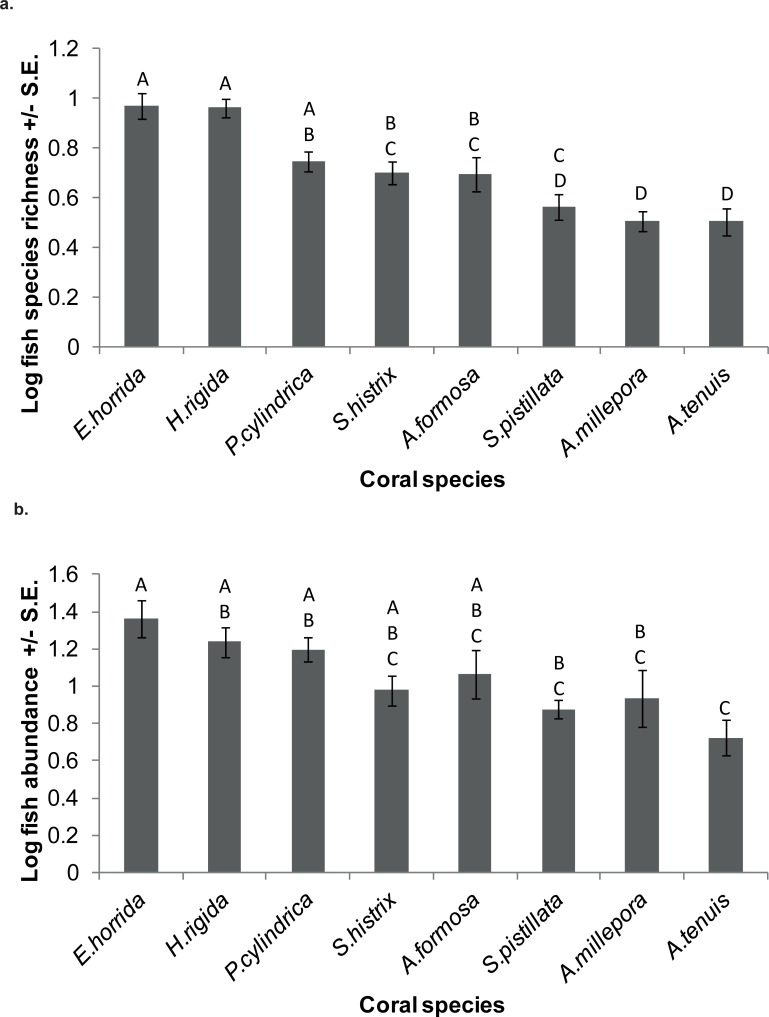
**(a)** Log fish species richness supported by eight coral species. Shared letters indicate no significant difference. **(b)** Log fish abundance supported by eight coral species. Shared letters indicate no significant difference.

Mean fish species richness differed among the eight coral species (F_7,70_ = 15.923, p < 0.001). A post hoc Tukey HSD test showed that *E*. *horrida* and *H*. *rigida* supported significantly higher fish species richness than the other coral species, with the exception of *P*. *cylindrica* (p < 0.05) (Fig 2A). *A*. *tenuis* and *A*. *millepora* colonies supported significantly lower fish species richness than most other coral species, with the exception of *S*. *pistillata* (Tukey HSD, p < 0.05) (Fig 2A). There was no significant difference in the fish species richness supported by *S*. *pistillata*, *S*. *hystrix and A*. *formosa* (Fig 2A).

There was a significant difference in the mean abundance of fish on the eight coral species (F_7,70_ = 5.015, p < 0.001). *E*. *horrida* supported a greater abundance than *A*. *tenuis*, *A*. *millepora* and *S*. *pistillata* (p < 0.05, Fig 2B). *A*. *tenuis* colonies supported significantly lower fish abundance than *E*. *horrida*, *H*. *rigida* and *P*. *cylindrica* colonies (p < 0.05) (Fig 2B). There was no significant difference in fish abundance among *A*. *millepora*, *A*. *formosa*, *H*. *rigida*, *P*. *cylindrica*, *S*. *hystrix* and *S*. *pistillata* (Fig 2B).

#### Fish assemblages

A total of 57 fish species and 1,205 individuals were observed occupying the eight coral species sampled at the 0.5 x 0.5 m spatial scale. Fish communities were dominated by damselfishes, which represented 47% (27 species) of the total number of species and just under 70% of the abundance of all individuals recorded, with *Chromis viridis* and *Pomacentrus moluccensis* being the two most abundant species.

**Fig 3:**
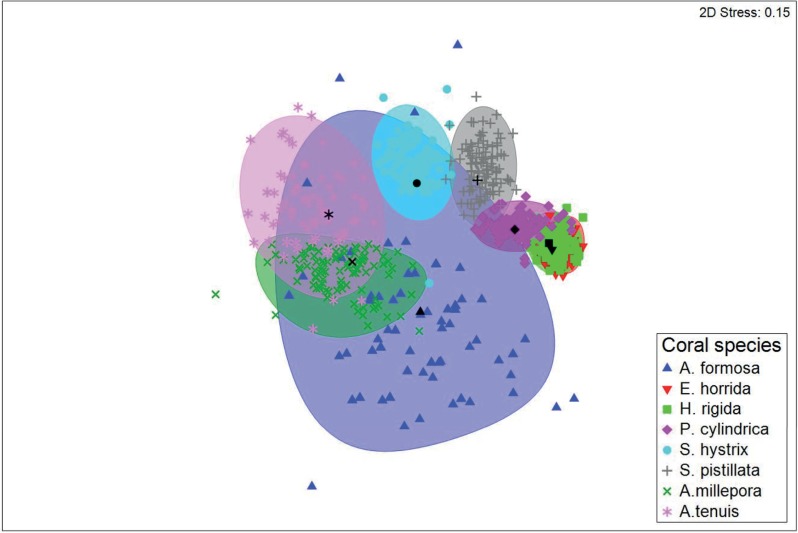
Two-dimensional, non-metric multidimensional scaling (nMDS) plot showing ordination of fish assemblages in relation to eight study coral species based on bootstrapped values calculated for 100 replications per coral species. Bootsrap averages (coloured symbols), group averages (black symbols) and 95% confidence intervals (coloured ellipses) based on bootstrap sampling with replacement are also shown.

Multivariate analysis of fish community composition revealed significant differences in fish assemblages among the eight coral species (PERMANOVA+ F_7,70_ = 4.1, p < 0.001; Fig 3). Pair–wise tests identified differences in fish community structure among most of the coral species examined; however, fish community structure did not differ between *E*. *horrida*, *H*. *rigida* and *P*. *cylindrica* or between *A*. *millepora*, *A*. *tenuis* and *A*. *formosa* (Table 2). These results were supported by SIMPER analysis, which showed high levels of dissimilarities between most coral species (~50–90%) (Table 3). *H*. *rigida* and *A*. *tenuis*, and *E*. *horrida* and *A*. *tenuis* had the most dissimilar fish communities (91% in both cases), whereas *E*. *rigida* and *H*. *horrida* had the most similar communities (52.4% dissimilarity). Bray-Curtis similarities for each coral species (within groups similarities) were relatively low ranging from 16% (*A*. *millepora*) to 49% (*E*. *horrida*). *C*. *viridis*, *P*. *moluccensis*, pomacentrid and labrid species exploited most of the studied corals. *Gobiodon histrio* was observed on *A*. *millepora* and *A*. *tenuis*, while *Paragobiodon xanthosomus* was only observed on *S*. *hystrix*. *N*. *melas* new settlers were predominantly observed on *A*. *millepora* and *A*. *tenuis*. While *S*. *nigricans* exclusively occupied *A*. *formosa* stands (Table 4).

**Table 2:** PERMANOVA pair-wise comparisons using Bray-Curtis similarity values to test for differences in fish community structure on different coral species.

Groups		t	p-value*	Unique permutations
***A*. *formosa***	*E*. *horrida*	2.1759	**0.0014**	4942
	*H*. *rigida*	2.2717	**0.0009**	5899
	*P*. *cylindrica*	1.8453	**0.0034**	4944
	*S*. *hystrix*	1.6432	**0.0064**	2886
	*S*. *pistillata*	1.6754	**0.0167**	3896
	*A*. *millepora*	1.2243	0.1904	1992
	*A*. *tenuis*	1.3958	0.1068	336
***E*. *horrida***	*H*. *rigida*	1.1394	0.264	9943
	*P*. *cylindrica*	1.2218	0.202	9916
	*S*. *hystrix*	2.668	**0.0001**	9872
	*S*. *pistillata*	2.341	**0.0002**	9885
	*A*. *millepora*	2.7335	**0.0001**	9797
	*A*. *tenuis*	3.0917	**0.0001**	7730
***H*. *rigida***	*P*. *cylindrica*	1.1824	0.2394	9935
	*S*. *hystrix*	2.579	**0.0001**	9905
	*S*. *pistillata*	2.29	**0.0003**	9930
	*A*. *millepora*	2.6789	**0.0001**	9857
	*A*. *tenuis*	3.0628	**0.0002**	8368
***P*. *cylindrica***	*S*. *hystrix*	2.0059	**0.001**	9870
	*S*. *pistillata*	1.716	**0.0099**	9904
	*A*. *millepora*	2.0693	**0.0001**	9763
	*A*. *tenuis*	2.1957	**0.0024**	7611
***S*. *hystrix***	*S*. *pistillata*	1.9203	**0.002**	9823
	*A*. *millepora*	1.7432	**0.0043**	9427
	*A*. *tenuis*	1.5334	**0.0405**	5704
***S*. *pistillata***	*A*. *millepora*	2.0047	**0.0005**	9660
	*A*. *tenuis*	1.8603	**0.0026**	6865
***A*. *millepora***	*A*. *tenuis*	0.62136	0.8354	4310

*Significant values are highlighted in bold.

**Table 3:** Results of SIMPER routine to analyse dissimilarity between groups (coral species). Pair-wise comparisons are shown.

	*A*. *formosa*	*E*. *horrida*	*H*. *rigida*	*P*. *cylindrica*	*S*. *hysrix*	*S*. *pistillata*	*A*. *millepora*
***A*. *tenuis***	82.8	91	91	85.2	80.6	82.9	75.2
***A*. *millepora***	84.6	89.4	88.6	87.2	87.7	89
***S*. *pistillata***	82.2	71.9	72.2	72.5	78.8
***S*. *hysrix***	84.7	78.5	78	77.6
***P*. *cylindrica***	82.5	59.5	59.9
***H*. *rigida***	80.4	52.4
***E*. *horrida***	77.7

**Table 4:** Percentage occurrence of fish taxa on each coral species.

	*A*. *formosa* (22%)	*E*. *horrida* (49%)	*H*. *rigida* (47%)	*P*. *cylindrica* (36%)	*S*. *hysrix* (28%)	*S*. *pistillata* (33%)	*A*. *millepora* (16%)	*A*. *tenuis* (25%)
Other NS	**13.6** **(34%)**	0.84	1.7	-	8	2.6	9.1	11.8
Other Juv	-	4.2	5.9	7.5	8	-	4.5	5.9
Pomacentrids	9.1	**8.4** **(22%)**	**9.3** **(25%)**	**10.4** **(20%)**	6	5.1	9.1	5.9
*Pomacentrus mollucensis*	4.5	**9.2** **(27%)**	**11** **(33%)**	**14.9** **(41%)**	8	**17.9** **(36%)**	4.5	-
*P*. *mollucensis* Juv*	9.1	4.2	1.7	7.5	**8** **(10%)**	**20.5** **(40%)**	9.1	**17.6** **(33%)**
*P*. *mollucensis* NS*	-	0.84	1.7	6	**10** **(14%)**	5.1	9.1	**17.6** **(26%)**
*Chromis viridis*	4.5	3.4	**6.8** **(10%)**	4.5	2	7.7	4.5	-
Labrids	9.1	**9.2** **(23%)**	5.9	6	2	7.7	-	-
*Gobiodon histrio*	-	-	-	-	-	-	**13.6** **(11%)**	11.8
*Paragobiodon xanthosomus*	-	-	-	-	**14** **(32%)**	-	-	-
*Neoglyphidodon melas* NS	4.5	-	-	-	-	-	**18.2** **(55%)**	**17.6** **(23%)**
*Stegastes nigricans*	**13.6** **(38%)**	-	-	-	-	-	-	-

* NS–new settlers; Juv–juveniles

Note: Those species that contributed >10% to the Bray-Curtis similarity of each group from SIMPER analysis are shown. The within group percent similarity is also displayed and individual species contribution to the within group percent similarities is given for major contributors (>10%) (in bold).

### Fish community structure and spatial scales of sampling

#### Fish species richness

**Fig 4:**
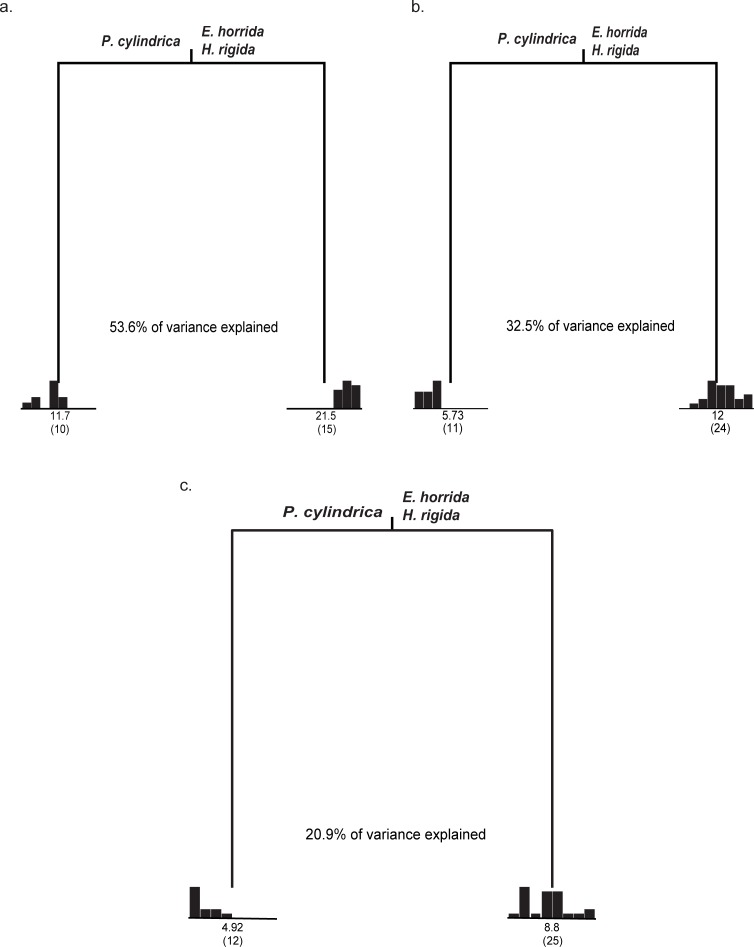
Regression tree analysis of the fish species richness at Lizard Island, Great Barrier Reef, QLD, Australia. The explanatory variables were: coral species, depth, and reef zone. For each of the terminal nodes the distribution of the observed values of fish species richness is shown in a histogram. Each node is labeled with the mean rating and the number of observations in a group (in parentheses). **(a)** 2x2 m scale. The tree explained 53.6% of the total variability in the data. **(b)** 1x1 m scale. The tree explained 32.5% of the total variability in the data. **(c)** 0.5x0.5 m scale. The tree explained 20.9% of the total variability in the data.

Coral species was the only variable that explained a significant amount of variation in fish species richness at each of the three sampling scales. Regression tree analyses for the three coral species sampled at the 2x2 m, 1x1 m and 0.5x0.5 m scale each produced a two-leaf tree (Fig 4). One leaf represented high fish species richness associated with *E*. *horrida* and *H*. *rigida* and the second leaf represented low fish species richness associated with *P*. *cylindrica* colonies. On average, *E*. *horrida* and *H*. *rigida* colonies contained twice as many fish species compared with *P*. *cylindrica* at each of the three sampling scales (Fig 4).

Overall, the largest sampling scale examined (2x2 m) explained twice as much of the variation in the data (53.6%) than the two smaller scales (32.5% and 20.9%, respectively). However, *E*. *horrida* and *H*. *rigida* supported a richer fish community than *P*. *cylindrica* regardless of the scale of sampling (Fig 4).

#### Fish abundance

**Fig 5:**
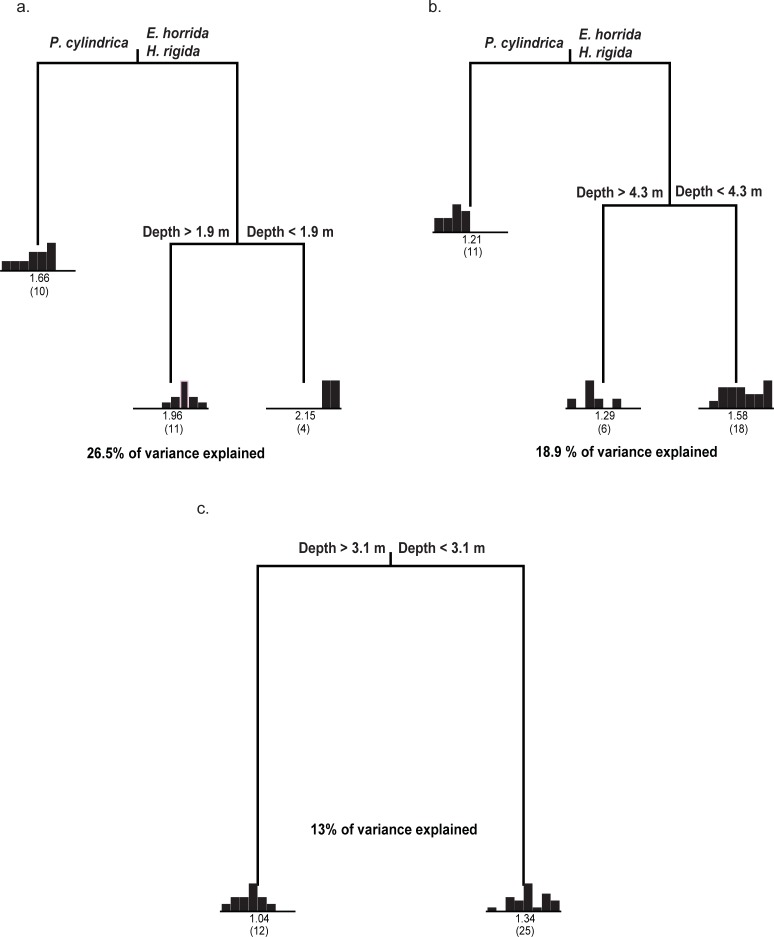
Regression tree analysis of the log fish abundance at Lizard Island, Great Barrier Reef, Australia. The explanatory variables were: coral species, depth, and reef zone. For each of the terminal nodes the distribution of the observed values of fish species richness is shown in a histogram. Each node is labeled with the mean rating and the number of observations in a group (in parentheses). **(a)** 2x2 m scale. The tree explained 26.5% of the total variability in the data. The first split based on coral species explained 15%, second split based on depth explained 11.5%. **(b)** 1x1 m scale. The tree explained 18.9% of the total variability in the data. The first split based on coral species explained 8.4%, second split based on depth explained 10.5%. **(c)** 0.5x0.5 m scale. The tree explained 13% of the total variability in the data.

Overall, as for species richness, the larger spatial scale explained much more variation in the abundance of fish species than the two smaller scales. Coral species explained a substantial amount of variation in fish abundance only at the two largest sampling scales (2x2 m and 1x1 m) (Fig 5). Regression tree analyses of log fish abundance for the three coral species sampled at the 2x2 m and 1x1 m scale produced a three-leaf tree in each case, explaining 26.5% and 18.9% of the variance, respectively (Fig 5). In both cases the first split was based on coral species, and the second split was based on water depth, explaining 15% (2x2 m, first split) and 11.5% (2x2 m second split), 8.4% (1x1 m, first split) and 10.5% (1x1m, second split) of the variation in fish abundance. One leaf represented high fish abundance associated with *E*. *horrida* and *H*. *rigida* colonies at shallower depth, the second leaf represented intermediate fish abundance associated with *E*. *horrida* and *H*. *rigida* colonies in deeper areas. The third leaf represented the lowest fish abundance associated with *P*. *cylindrica* colonies. On average, *E*. *horrida* and *H*. *rigida* colonies supported twice as high fish abundance as *P*. *cylindrica* at 2x2 m and 1x1 m scales (Fig 5A and 5B). The regression tree analysis of the log fish abundance for the three coral species sampled at the 0.5x0.5 m scale produced a two-leaf tree explaining 13% of the variance (Fig 5C).

#### Fish community structure and coral colony size

**Fig 6:**
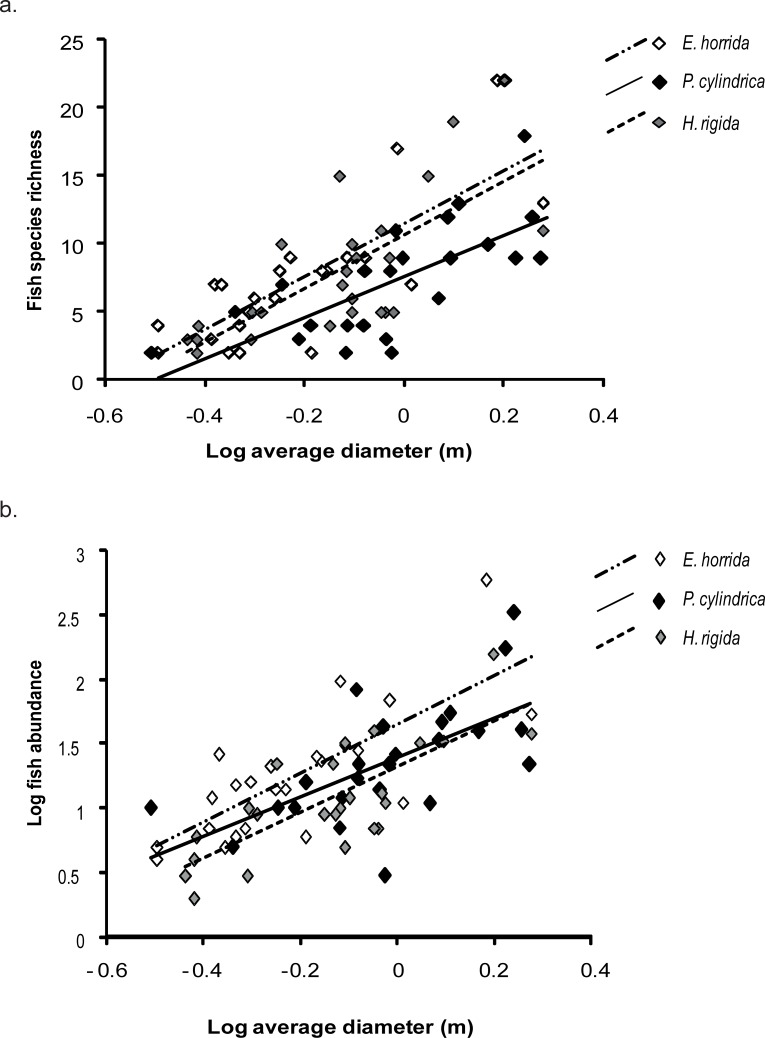
**(a)** Relationships between fish species richness supported by three different coral species and an average diameter of a range of coral colonies. **(b)** Relationships between fish abundance supported by three different coral species and average diameter of a range of coral colonies.

Fish species richness increased as colony size increased (ANCOVA; colony size F_1,67_ = 75.3195, p < 0.001; Fig 6A). There was no interaction between coral species and coral colony size (Homogeneity of slopes, F_2,65_ = 0.5147, p > 0.05) indicating that all three coral species accumulated fish species richness at approximately the same rate with increasing colony size. *E*. *horrida* and *H*. *rigida* supported higher fish species richness than *P*. *cylindrica* on colonies of similar size (ANCOVA; coral species, F_2,67_ = 6.1785, p < 0.05; Fig 6A).

Similarly, log fish abundance increased with colony size (ANCOVA; colony size, F_1,67_ = 70.6107, p < 0.001, Fig 6B). There was no interaction between coral species and colony size (Homogeneity of slopes, F_2,65_ = 0.2785, p > 0.05) indicating that all three coral species accumulated fish abundance at approximately the same rate with increasing colony size. *E*. *horrida* supported higher log fish abundance then *P*. *cylindrica* and *H*. *rigida* (ANCOVA; coral species, F_2,67_ = 4.6293, p < 0.05; Fig 6B).

## Discussion

Substantial differences in fish species richness, abundance and assemblage structure were found among the eight coral species. Two coral species, *E*. *horrida* and *H*. *rigida*, supported the highest fish species richness and abundance. In general, the different corals supported distinct fish communities, with dissimilarities ranging from 50–90%. Coral species was the main variable explaining variation in fish species richness at three different sampling scales, with more variation explained at the largest scale of sampling. Fish species richness and abundance increased with coral colony size at similar rates for different coral species, but there were substantial differences among species at similar coral colony sizes. These results have important implications for interpreting the effects of declining coral cover, species diversity and coral size in response to the increasing severity and frequency of disturbances impacting on coral reefs.

### Importance of coral species

Our results are consistent with other studies showing that coral diversity has a strong influence on fish species richness and abundance [25, 41]. Our findings also indicate that some coral species tend to support more diverse and abundant fish communities than other coral species and therefore may play a more important role in supporting fish diversity. *E*. *horrida* and *H*. *rigida* supported richer and more abundant fish communities than the other six coral species examined. Three coral species *A*. *millepora*, *A*. *tenuis* and *S*. *pistillata* supported the least diverse and least abundant fish communities. Several studies have investigated the importance of different *Acropora* and *Pocillopora* species as coral reef fish habitat [23, 27, 42], however other common coral species, which may be important fish habitat, have received less attention. While there is an obvious effect of coral species identity on fish communities, there may also be an interaction effect of different coral species, with some combinations being able to support higher fish diversities and abundances. These possible relationships require further investigation.

Current estimates indicate that less than 10% of reef fish species are coral dependent [18, 24], although a much larger proportion of species are known to respond to declining coral cover [8, 42]. The strength of the species-specific patterns observed here suggest a large number of small fish species discriminate among coral species in some way. Many fish species prefer to settle on or near live coral [23–24, 43], even if adults of the same species are not coral dependant. Coker et al. [24] reported that there is large variation in habitat specialisation on coral reefs, with some fish species being strongly linked to single coral species and others being found to occupy a number of species of corals. The corals in our study that supported the highest richness of fishes appeared to be providing suitable habitat for a large array of fish species.

Differences in fish species richness, abundance and community composition supported by different coral species may potentially be related to the branching structure of the corals. In general, coral species with an intermediate branch spacing and length supported the most rich and abundant fish communities (e.g. *E*. *horrida*, *H*. *rigida*). Small fish species or younger life stages were predominantly associated with tightly branched corals like *A*. *millepora* and *A*. *tenuis*, while the large damselfish *S*.*nigricans* was almost exclusively confined to *A*. *formosa* coral colonies, which was the most open branching coral in this study. Although branching complexity does not necessarily exclude predators, it is likely to aid in prey escape [44]. Tightly branched corals would be expected to decrease predation levels and allow higher survival for smaller fish species; however, only a few fish species would be able to use these corals as refuge due to the size limitations (e.g. corymbose corals, such as *A*. *millepora*, *A*. *tenuis*). On the other hand, the more open corals, with larger distances between branches and abundant free space available, would allow a large number of different fish species to enter the colony; however, it also means that larger predators can access prey more easily. Coral species that provide adequate space among the branches for movement and feeding of resident fish, and at the same time a sufficiently dense structure to offer protection from larger predators, might be expected to be favoured by a wide range of small reef fishes. Coral reef fish often use holes of approximately their own body diameter as shelter [21, 27], which may explain why more structurally complex coral species support more diverse fish communities.

Although coral species was the most important factor affecting the structure of fish communities, water depth also had a small, but detectable, influence on overall fish abundance. Water depth explained 10–15% of the variance in fish abundance, with higher abundances associated with coral colonies in shallower water. It has been suggested that the relationship between fish abundance and live coral cover may be stronger in shallower zones, as fish are forced to remain in close proximity to the substratum in such areas [45].

### Importance of sampling scale

Coral species explained a greater amount of total variation in fish species richness and fish abundance at the largest sampling scale examined (2x2 m) compared with two smaller sampling scales. Moreover, while coral species explained a relatively large amount of variation in fish species richness at all three sampling scales (21–54%), this relationship was not as strong for fish abundance (0–15%). The findings indicate that fish species richness-habitat associations become less apparent at very small spatial scales. The smaller change in the proportion of variation in fish abundance explained at all three spatial scales, compared with fish species richness, indicates that spatial scale has less influence on fish abundance-habitat associations than it does on fish species richness-habitat associations. These results show that the spatial scale of sampling can have a significant effect on the strength of the relationship between coral and fish communities, but does not alter the basic patterns.

### Importance of colony size

We found that fish species richness and abundance increased with coral colony size for the three coral species examined. Other studies have also reported positive correlations between reef or coral head size and corresponding fish species richness or fish abundance [21, 46–47]. This relationship is usually explained by the assumption that larger areas contain larger numbers of refuges and are likely to create a greater number of microhabitats. Therefore, larger areas can facilitate niche partitioning and support a greater number of individuals, and thus, larger areas are also likely to contain a larger number of species [21, 48–49]. Fish abundance and richness increased at a similar rate with increasing colony size for the three coral species examined. This suggests that the same mechanisms may be regulating abundance and species richness on the three coral species. One such mechanism could be coral structural characteristics. In the lagoon of Lizard Island, the variations in fish species richness and fish abundance were explained by coral species themselves. *E*. *horrida* and *H*. *rigida* supported higher fish species richness than *Porites cylindrica* at all sizes, while *E*. *horrida* supported higher fish abundance than the other two corals at all sizes.

### Conclusion

This study found that the diversity and abundance of the fish communities was strongly related to the coral species examined. Furthermore, the strength of the association with coral species was stronger for species that could be sampled at larger spatial scales. The majority of fish species exhibited preferences for two of the coral species surveyed, *E*. *horrida* and *H*. *rigida*. In addition, for a given coral species, fish species richness and abundance increased as colony size increased, however *E*. *horrida* and *H*. *rigida* supported higher fish species richness than *P*. *cylindrica* at all colony sizes. These results suggest that similar processes influence fish distribution up to the largest scale examined here and that physical characteristics of the coral species are likely to have a significant influence on both the number of individuals and the number of fish species found on coral colonies.

These results have important implications for our understanding of the likely effects of degrading coral communities on fish communities. Often, the coral species supporting the most diverse fish communities also appear to be highly susceptible to coral bleaching, storms and cyclones, and other disturbances [23, 32–33]. Various disturbances will tend to reduce the average size of surviving coral colonies, further negatively influencing the abundance and diversity of coral reef fish communities. Together, these results suggest that a reduction in the cover of coral species, especially those that support diverse and abundant fish communities, could cause significant reductions in the diversity and abundance of local coral-associated fish communities. Triggers to initiate management actions are often based on gross estimates of declining coral cover. However, management plans to preserve the biodiversity of coral reefs must focus on detecting declines in and protecting structurally complex coral species.

## Supporting information

S1 Supporting Information Assumptions testing. (DOCX)
