# Supplementary material for: Strong effects of coral species on the diversity and structure of reef fish communities: A multi-scale analysis
Source: PLoS One. 2018 Aug 13;13(8):e0202206. doi: 10.1371/journal.pone.0202206 (PMC6089460; doi:10.1371/journal.pone.0202206)
Supplement: S1 Supporting Information — (DOCX) [file pone.0202206.s001.docx]

### Section A. Coral structural characteristics.

Branch length was log10 transformed to meet the ANOVA assumptions of normality and homoscedasticity. The mean of ten inter-branch spaces (eight coral species) was also calculated for each coral colony before performing nonparametric independent samples Kruskal-Wallis test, as the data did not meet the assumptions for ANOVA.

*Branch length (log10)*

Normality


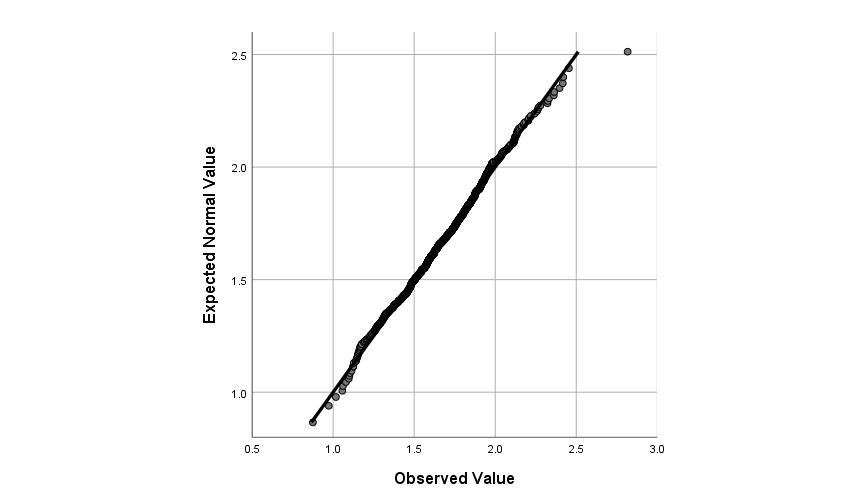


S1 Fig 7. Normal Q-Q plos for log10 transformed coral branch length.

Homogeneity of variances

Levene’s statistics_5,294_ = 1.107, p = 0.355

*Inter-branch space (log10)*

Normality


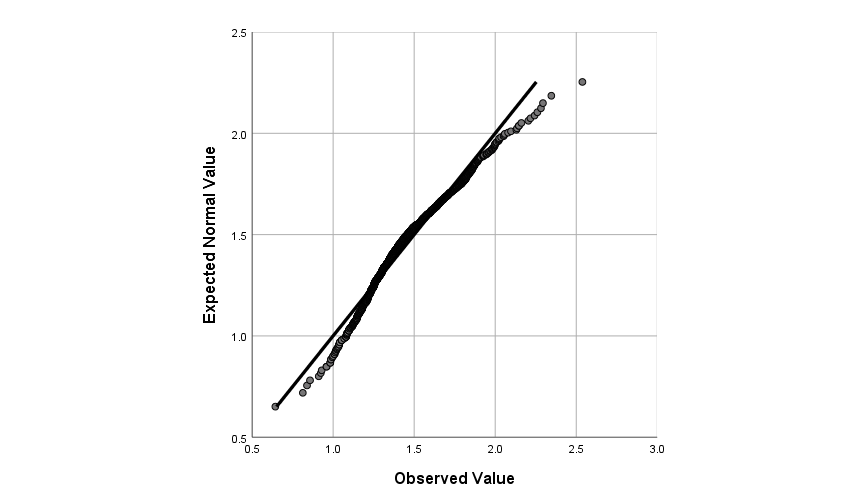


S1 Fig 8. Normal Q-Q plos for log10 transformed coral inter-branch space.

Homogeneity of variances (violated)

Levene’s statistics_7,1222_ = 24.927, p = 0.000

### Section B. Fish community structure and coral species.

Fish species richness and fish abundance were log10 transformed to meet the ANOVA assumptions of normality and homoscedasticity.

*Fish species richness (log10)*

Normality


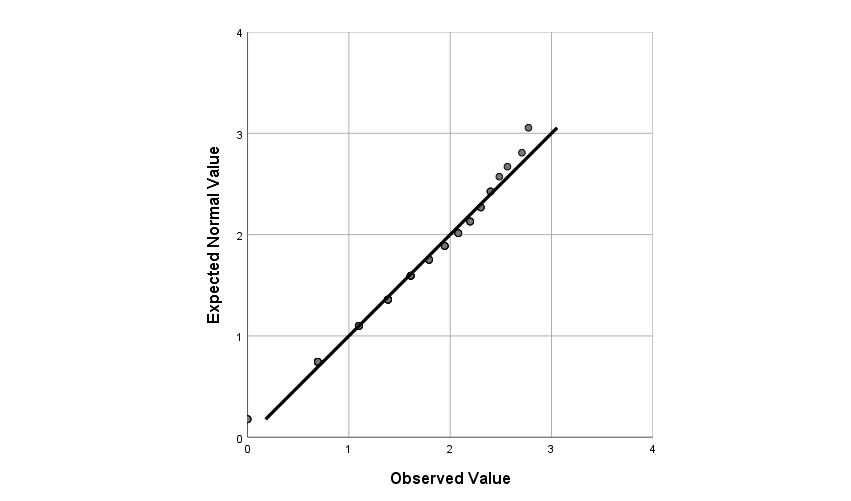


S1 Fig 9. Normal Q-Q plos for log10 transformed fish species richness.

Homogeneity of variances

Levene’s statistics_7,70_ = 0.823, p = 0.572

*Fish abundance (log10)*

Normality


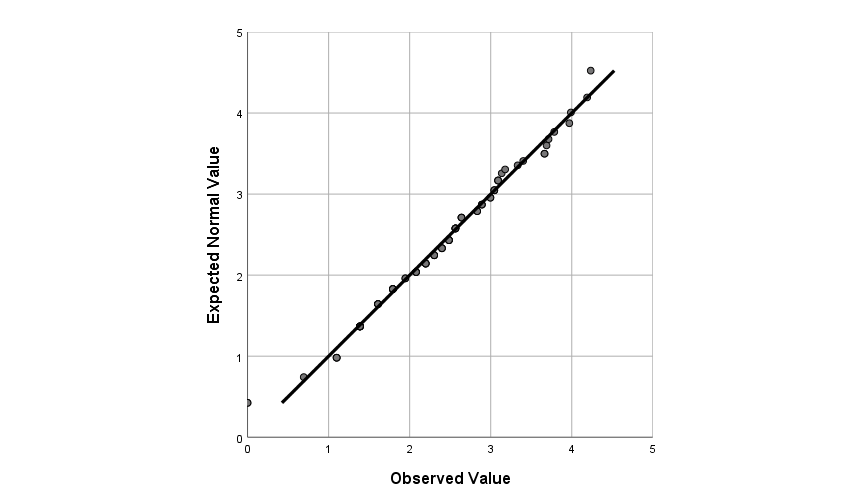


S1 Fig 10. Normal Q-Q plos for log10 transformed fish abundance.

Homogeneity of variances

Levene’s statistics_7,70_ = 2.062, p = 0.059

**Section C. Fish community structure and spatial scales of sampling.**

Fish abundance was log10 transformed to reduce the influence of extreme values.

*Fish species richness*


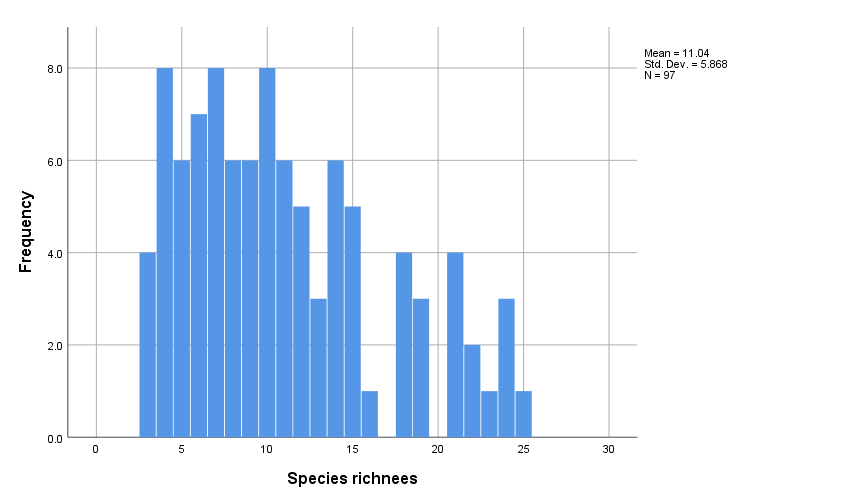


S1 Fig 11. Histogramm of the frequency distribution of fish species richness counts. The data is normally distrubuted without any extreme values.

*Fish abundance*

a.


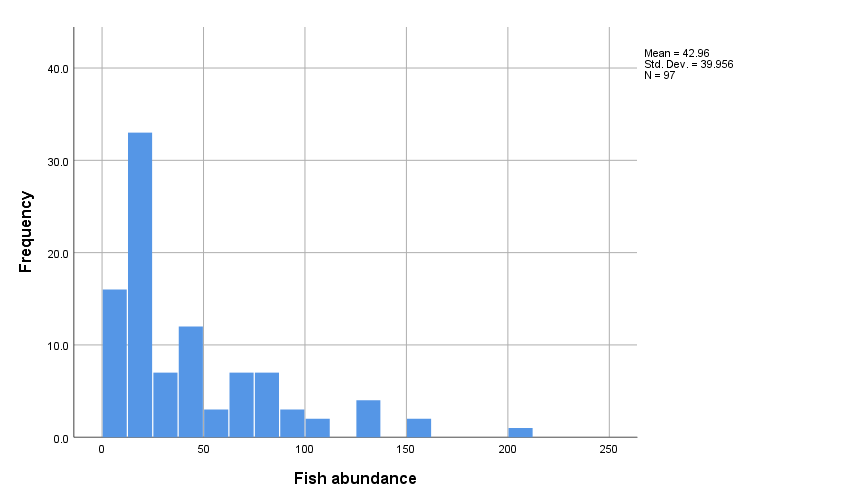


b.


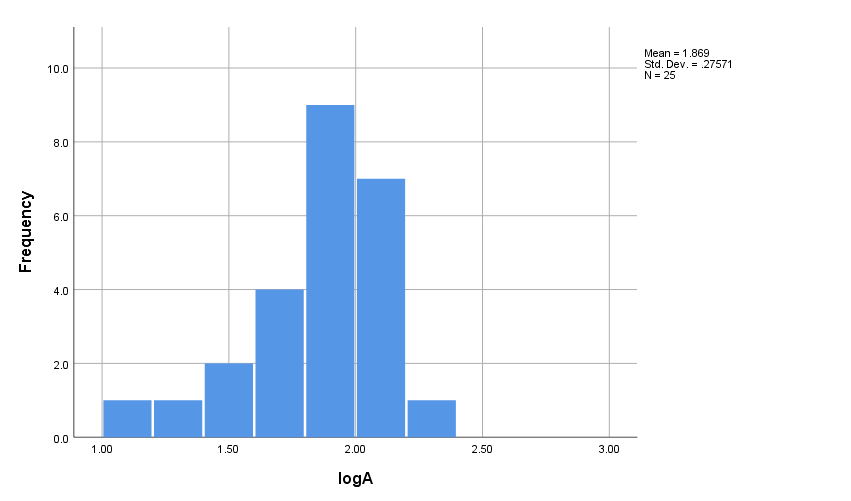
S1 Fig 12. Histogramm of the frequency distribution of fish abundance counts. a. Untransformed. The distribution clearly shows presence of a few very high counts in the data. b. log10 transformed distribution shows the reduction of the extreme values.

### Section D. Fish community structure and coral colony size.

Fish abundance and colony size (expressed as colony average diameter) were log10 transformed to meet the assumptions of normality, homoscedasticity and linearity.

*Fish species richness*

Normality

a.


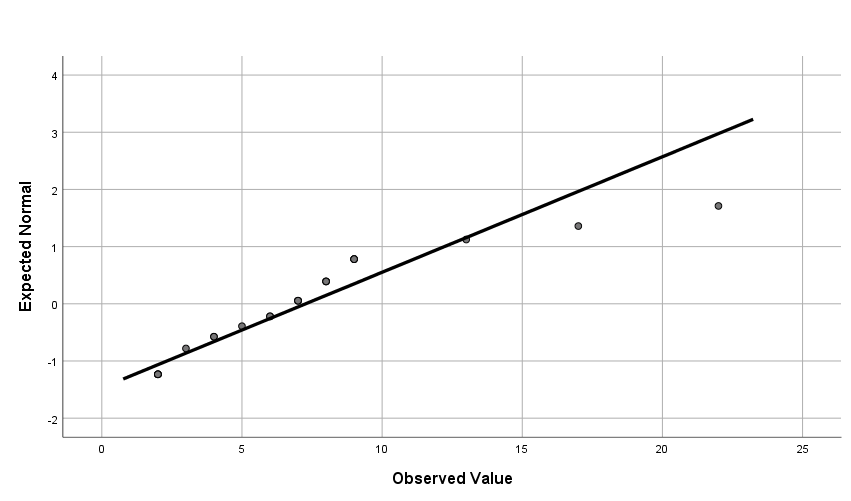


b.


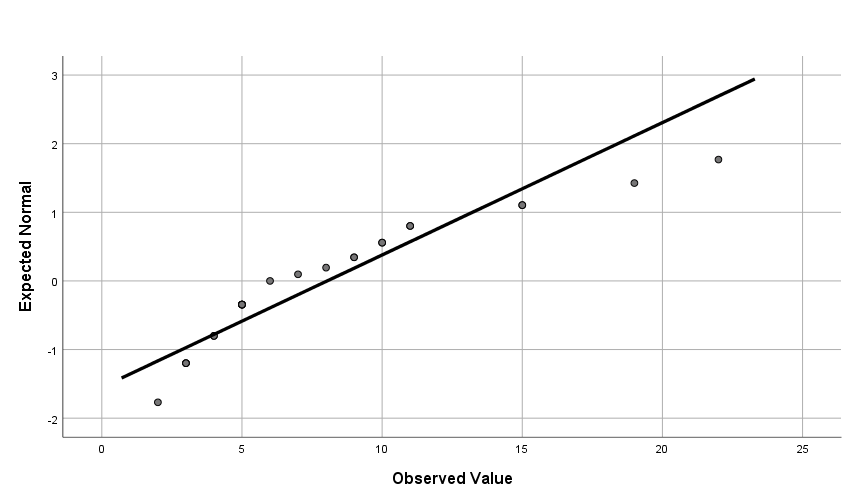


c.


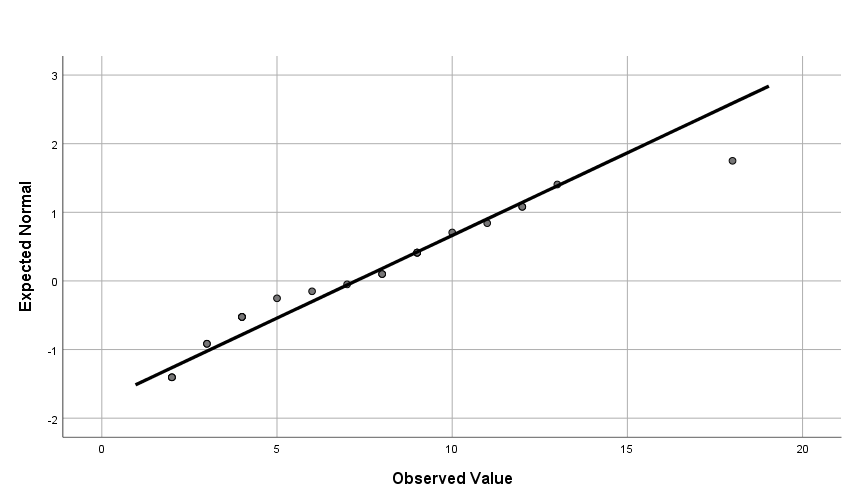


S1 Fig 13. Normal Q-Q plos for fish species richness. a. *E. horrida* b. *H. rigida* c. *P. cylindrica*

Homogeneity of variances

Levene’s statistics_2,68_ = 0.405, p = 0.669

Design: intercept + coral species + average diameter (log10)

Linearity


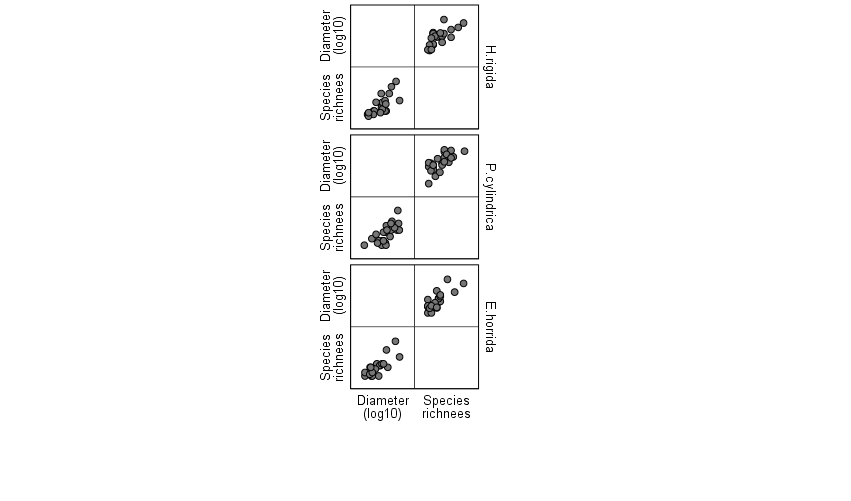


S1 Fig 14. Scatterplot for fish species richness and average diameter of the coral colony (log10) for each coral species examined.

*Fish abundance (log 10)*

Normality

a.


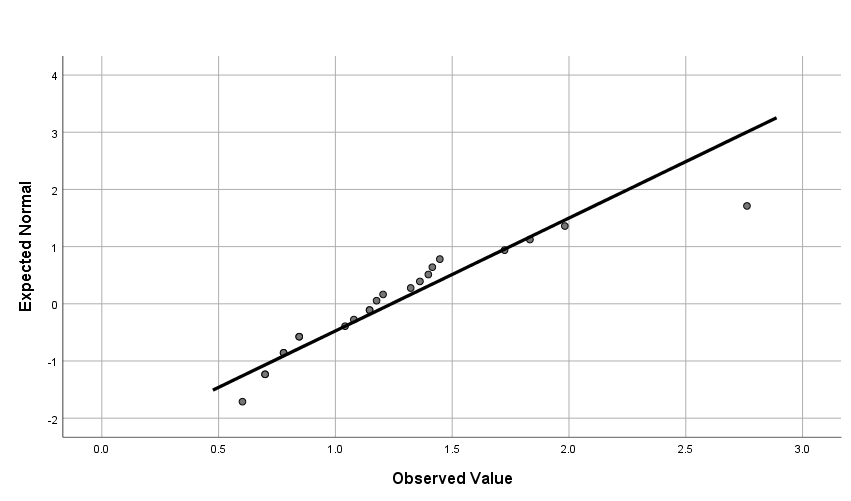


b.


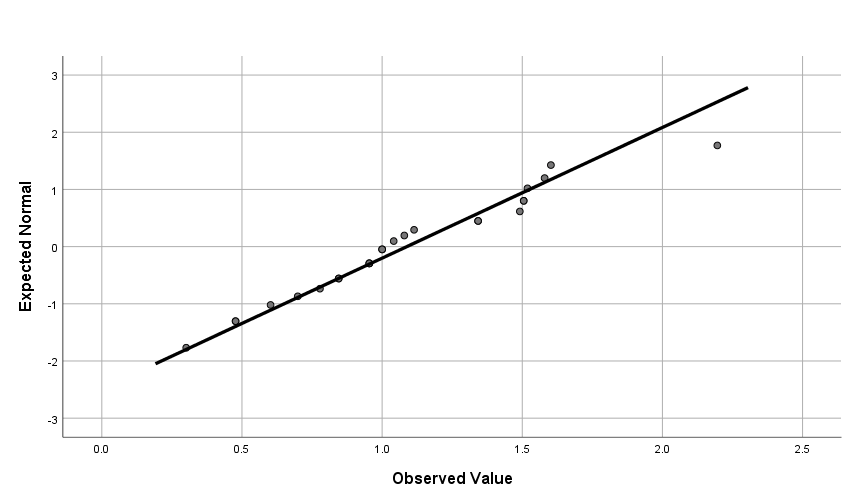


c.


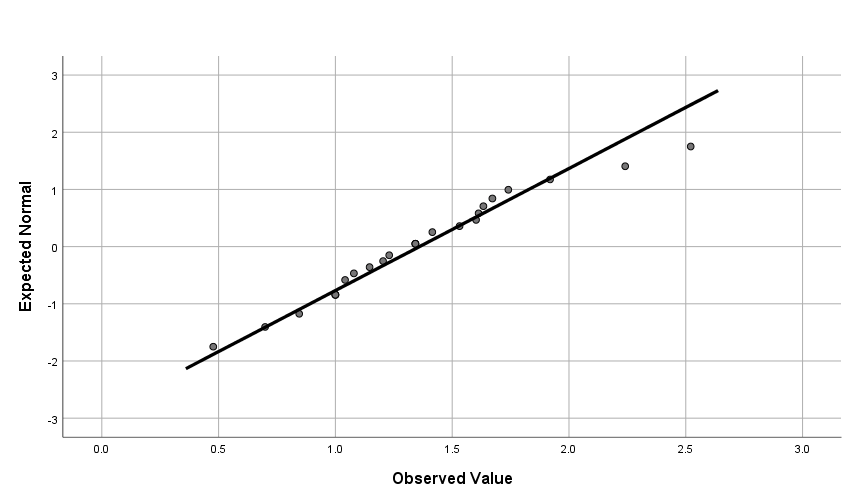


S1 Fig 15. Normal Q-Q plos for log10 transformed fish abundance. a. *E. horrida* b. *H. rigida* c. *P. cylindrica*

Homogeneity of variances

Levene’s statistics_2,68_ = 0.055, p = 0.946

Design: intercept + coral species + average diameter (log10)

Linearity


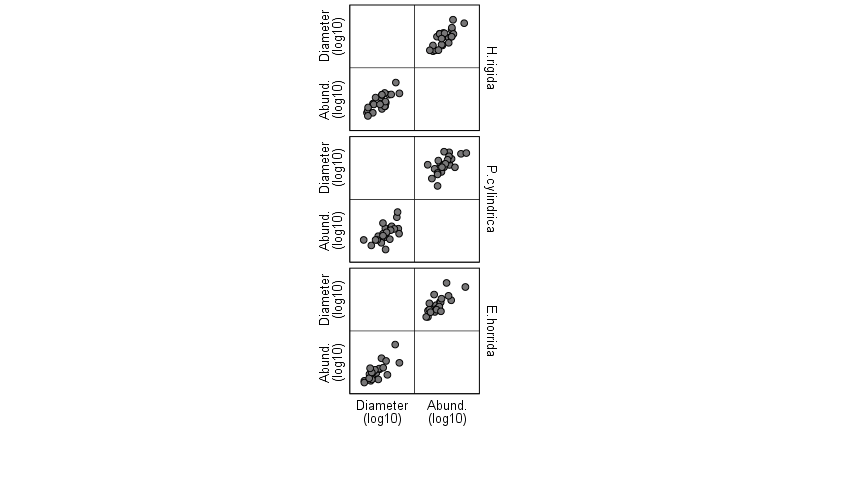


S1 Fig 16. Scatterplot for fish abundance (log10) and average diameter of the coral colony (log10) for each coral species examined.
